# Supplementary material for: Octogenarians’ Breast Cancer Is Associated with an Unfavorable Tumor Immune Microenvironment and Worse Disease-Free Survival
Source: Cancers (Basel). 2021 Jun 11;13(12):2933. doi: 10.3390/cancers13122933 (PMC8230790; doi:10.3390/cancers13122933)
Supplement: Supplementary file 1 [file cancers-13-02933-s001.zip › cancers-1219016-supplementary.pdf]

Supplementary Material: Octogenarians' Breast Cancer Is Associated with an Unfavorable Tumor Immune Microenvironment and Worse Disease-Free Survival

Maiko Okano, Masanori Oshi, Swagoto Mukhopadhyay, Qianya Qi, Li Yan, Itaru Endo, Toru Ohtake and Kazuaki Takabe

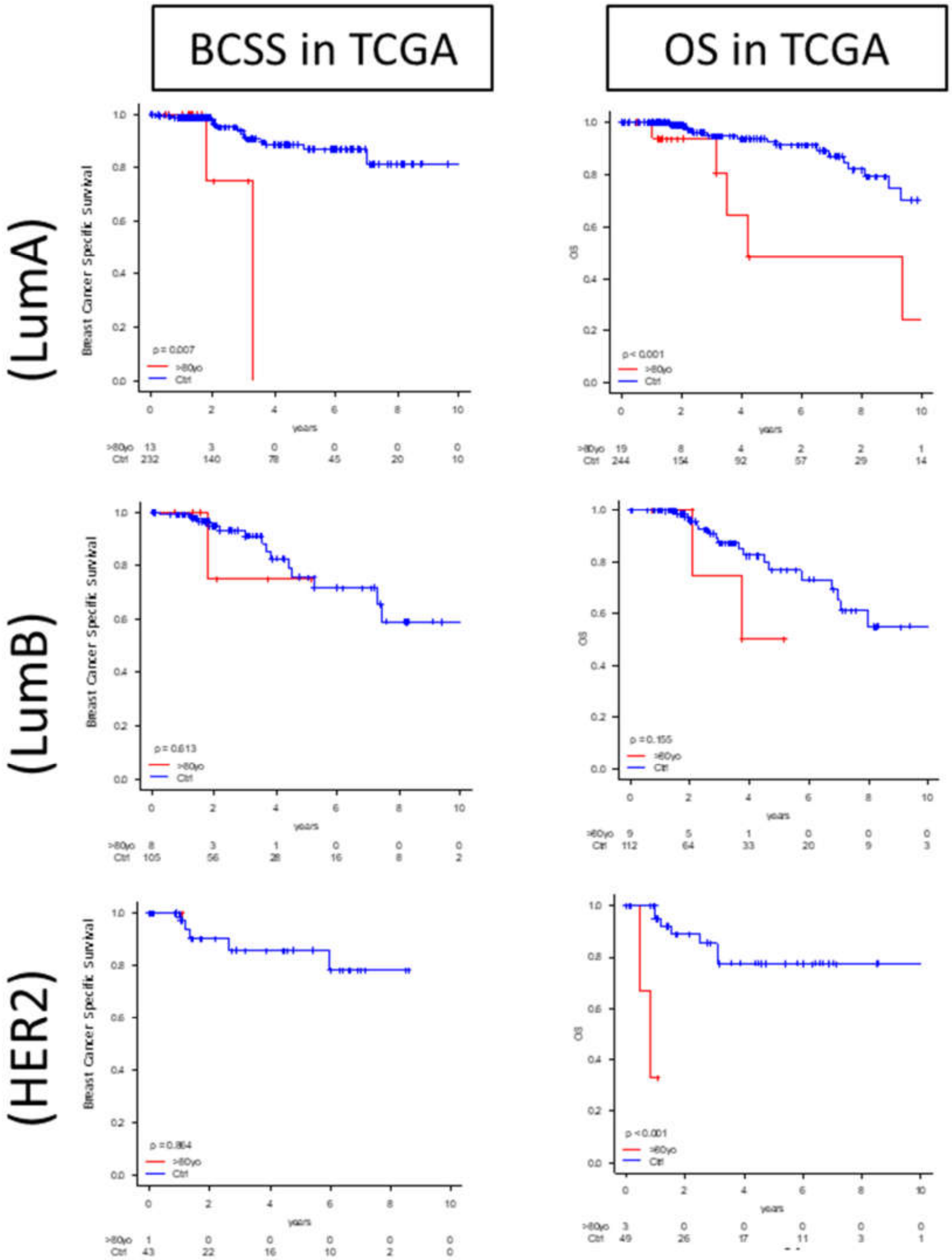

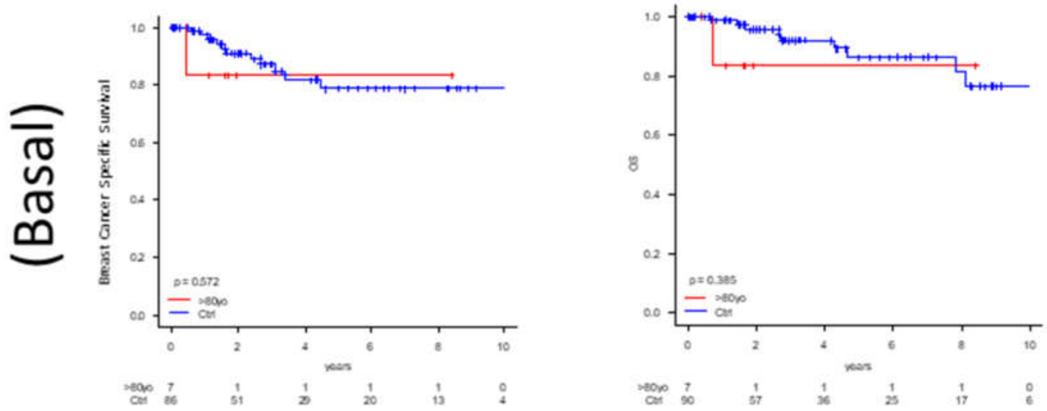

Figure S1. Kaplan–Meier survival curves of breast cancer specific survival (BCSS) and overall survival (OS) analyses between the Octogenarian (red line) and the control group (blue line) by subtypes in TCGA cohort.

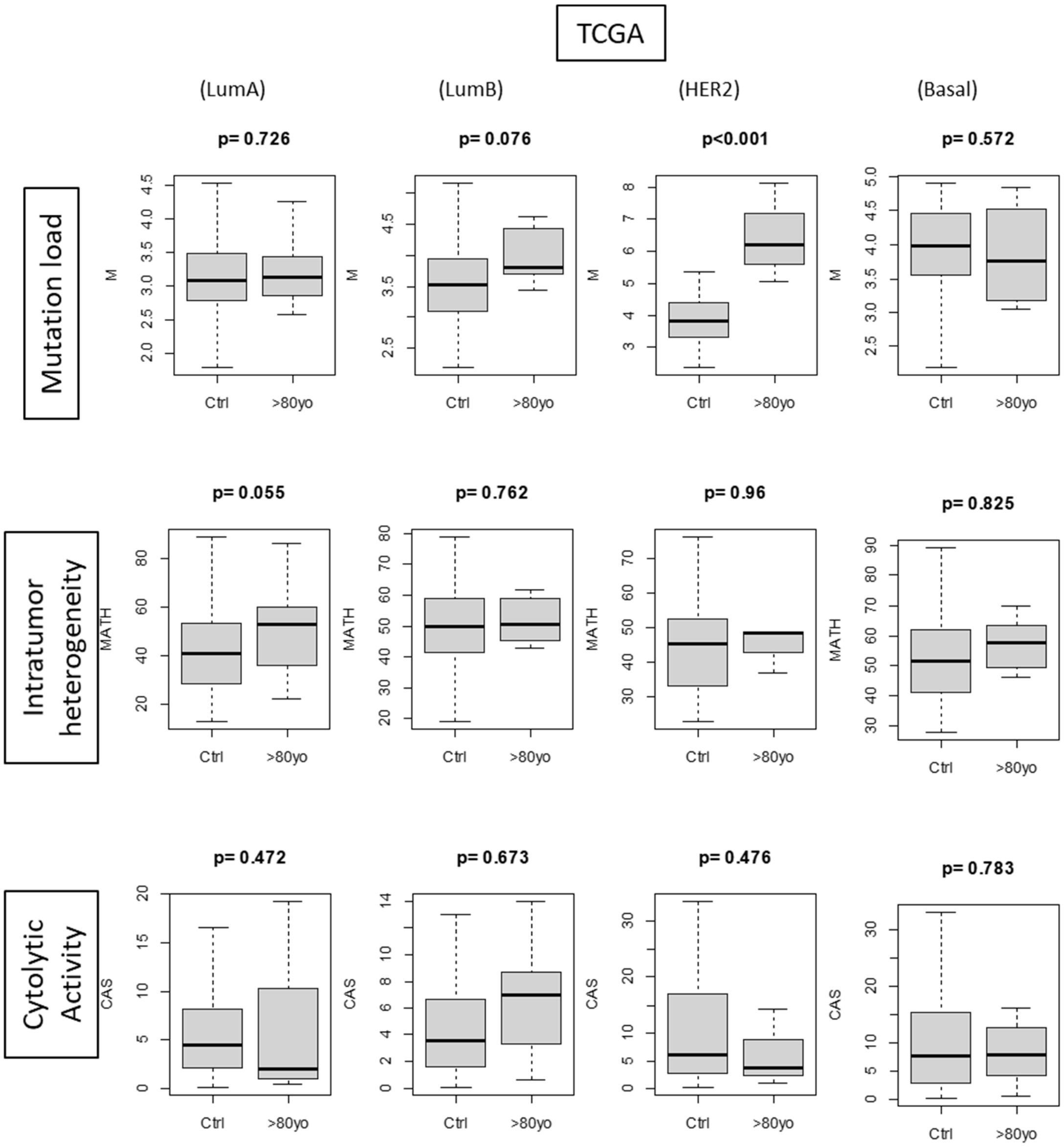

Figure S2. Boxplots of comparison of the control group and the Octogenarians in mutation load, Intratumor heterogeneity (MATH score), and Cytolytic Activity Score (CYT) by the subtypes in TCGA cohort.

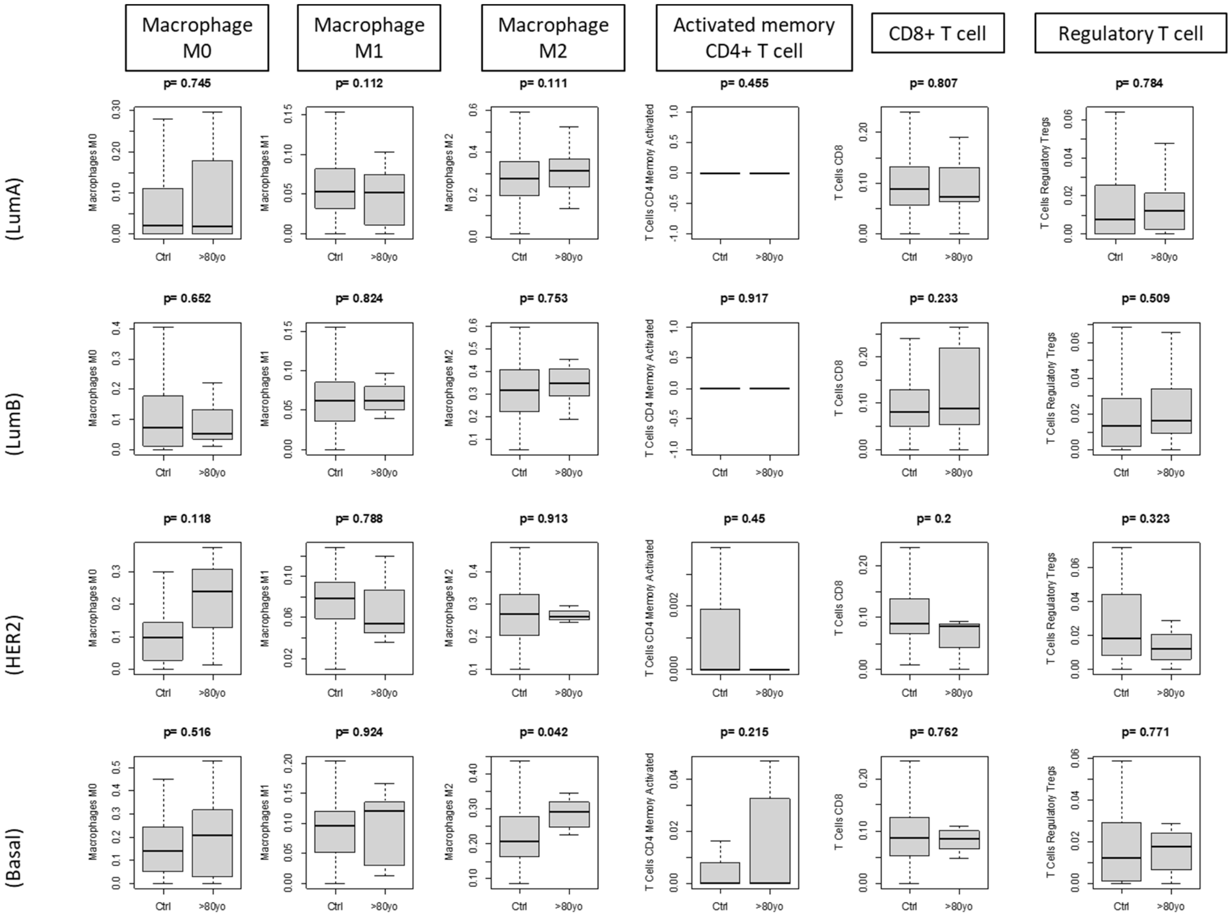

Figure S3. Fraction of Macrophage (M0), M1, M2 activated memory CD4 T cell, CD8 T cell and Regulatory T cell in control group and Octogenarian by the subtypes in TCGA cohort.
